# Ethical Conflicts and Knowledge of the Code of Ethics Among Occupational Therapists in Spain

**DOI:** 10.3390/healthcare14030367

**Published:** 2026-01-31

**Authors:** Daniel Emeric-Méaulle, Pablo A. Cantero-Garlito, Ana A. Laborda-Soriano

**Affiliations:** 1 Faculty of Health Sciences, Universidad Rey Juan Carlos, 28922 Alcorcón, Spain; daniel.emeric@urjc.es; 2 Faculty of Health Sciences, Universidad de Castilla–La Mancha, 45600 Talavera de la Reina, Spain; 3 Faculty of Health Sciences, Universidad de Zaragoza, 50009 Zaragoza, Spain; analabor@unizar.es

**Keywords:** professional ethics, occupational therapy, code of ethics, ethical conflict, ethical reasoning

## Abstract

**Objective:** This study characterized Spanish occupational therapists’ knowledge of the national Code of Ethics and perceptions of professional ethics and examined associations with sociodemographic and educational variables. It quantified knowledge of key Code elements (approving body and professional values), described ethics education and participation in formal ethical support structures, and identified resources used to manage ethical conflicts in routine practice. **Methods:** A descriptive cross-sectional online survey was administered between March and September 2022. The analytical sample included 596 occupational therapists practicing in Spain. The questionnaire assessed participant characteristics, ethics education, knowledge and perceived importance of the Code, participation in ethics committees or similar structures, experience of ethical conflicts, and conflict-management strategies. Descriptive and bivariate analyses were conducted (*p* < 0.05). **Results:** Respondents were mostly women (86.6%) and aged 20–40 years. Although 65.3% reported university ethics education and 73.2% rated the Code as important/very important, 11.4% were unaware of its existence. Only 28.2% identified the approving body, and 16.3% correctly identified the professional values included in the Code. Ethical conflicts were reported by 43.1%. When conflicts occurred, respondents most often consulted the interdisciplinary team (25.5%) or occupational therapy colleagues (24.3%), whereas few consulted the Code (4.5%) or an ethics committee (2.7%). Ethics education and greater professional experience were associated with higher Code knowledge. **Conclusions:** Occupational therapists in Spain endorse professional ethics, yet actionable knowledge and use of the Code and engagement with formal support structures remain limited. Strengthening practice-oriented ethics education and accessible deliberation mechanisms may improve ethical decision-making.

## 1. Introduction

Ethics is one of the fundamental pillars of health professions, guiding professional action toward respect, autonomy, and justice [1]. In the field of occupational therapy, ethical dimensions acquire particular relevance due to the relational and deliberative nature of practice, which requires balancing clients’ needs, values, and rights with the institutional, economic, and social constraints that shape intervention contexts [2,3,4]. This balance between the subjective dimensions of doing, ethical principles, and structural conditions illustrates the moral complexity of occupational therapy practice and situates ethics as a constitutive component of the occupational therapist’s clinical reasoning [5,6].

International studies have documented the presence of ethical tensions derived from value conflicts, inequities in resource distribution, and discrepancies between institutional policies and the principles of person-centered care [2,3,4,7]. These tensions frequently manifest as moral dilemmas or as moral distress, understood as recognizing the ethically appropriate action but being unable to carry it out due to external, hierarchical, or systemic constraints [8,9]. Moral distress affects not only the emotional well-being of professionals but also the quality of care and their capacity to sustain a practice grounded in justice and human dignity [10,11].

In this context, ethical and deontological codes constitute essential instruments for guiding responsible decision-making and safeguarding the integrity of professional practice [2,12]. In Spain, the Occupational Therapy Code of Ethics, approved by the General Council of Professional Associations of Occupational Therapists of Spain (CGCTO) in 2020, establishes the principles and values that should guide professional conduct, aiming to protect both clients and therapists [13]. However, the mere existence of a code does not guarantee its knowledge or effective implementation, especially in settings characterized by recently established professional structures, as is the case of Spanish occupational therapy. International research has shown the gap that may exist between normative frameworks and everyday ethical practice [14,15], emphasizing that ethical reasoning requires not only normative references but also moral deliberation skills and institutional environments that support ethical reflection [2,5].

The literature indicates that ethical reasoning in occupational therapy is shaped by academic training, professional experience (including career trajectory, practice settings, and populations served), and organizational culture. Laliberté et al. [6] highlighted the heterogeneity of ethics education in physiotherapy and occupational therapy programs in Canada, underlining the need to integrate ethical reflection in a transversal and practical manner. Similarly, Drolet [16] stresses the importance of articulating theory and practice in applied ethics, promoting epistemic humility and recognizing context as a determining factor in moral judgment.

In Spain, available empirical evidence suggests heterogeneous familiarity with the national Code of Ethics—ranging from general awareness of its existence to limited knowledge of its approving body, core principles, and reported use in practice [5]. This unevenness is relevant because the Code is intended to function not only as a symbolic reference but also as an operational guide to support ethically justified clinical reasoning in everyday situations. When institutional mechanisms for deliberation and guidance are scarce, ethical problems are more likely to be managed individually through personal judgment or informal consultation, which can increase variability in decision-making and contribute to drift from shared professional norms (e.g., consent processes, confidentiality boundaries, and prioritization under resource constraints) [2,5,17]. In such contexts, professionals may rely on tacit norms within local teams or on personal experience, rather than on explicitly articulated principles, which can hinder consistent practice and reduce opportunities for collective learning. This gap between symbolic endorsement and practical, operational knowledge underscores the need to examine how occupational therapists access, interpret, and apply the Code in routine clinical reasoning and how these patterns relate to training opportunities and workplace contexts [2,12].

Therefore, the aim of this study was to characterize Spanish occupational therapists’ knowledge of the national Code of Ethics and their perceptions of professional ethics and to examine their associations with sociodemographic and educational variables. Specifically, we sought to: (1) quantify knowledge of key elements of the Code (e.g., approving body and core principles); (2) describe ethics education and involvement in formal ethical support structures; and (3) identify the resources and strategies used to address ethical conflicts in daily practice.

## 2. Materials and Methods

### 2.1. Study Design

A descriptive, observational, and cross-sectional study was conducted to explore the level of knowledge and application of the Occupational Therapy Code of Ethics, as well as the experiences of ethical conflicts and the strategies used for their resolution among professionals in Spain. This type of design was considered appropriate to obtain a general overview of the current situation, identify trends, and establish relationships among sociodemographic, educational, and ethical variables [18].

### 2.2. Participants and Sample

The target population consisted of licensed occupational therapists actively practicing in Spain. The inclusion criteria were (a) holding a university degree in Occupational Therapy (Diploma or Bachelor’s/Graduate degree), (b) currently practicing occupational therapy in Spain, and (c) being under 65 years of age.

The sample size was estimated using the total number of registered occupational therapists in Spain (*n* = 6788) as of December 2021 [19]. An a priori calculation for a finite population, assuming a 95% confidence level and a 5% margin of error, indicated a minimum required sample of 364 participants. This threshold was exceeded, as the final sample comprised 596 respondents.

A non-probabilistic convenience sampling strategy was used through the collaboration of regional professional associations, occupational therapy networks, and social media. This strategy enabled broad and heterogeneous participation, covering different autonomous communities and work settings.

### 2.3. Instrument

Data were collected using an ad hoc structured questionnaire developed from a systematic review of the literature on professional ethics in occupational therapy [2,3,4,7,8,9,17] and reviewed by a panel of five expert occupational therapists specializing in teaching, clinical management, and applied ethics.

The questionnaire included five main sections:

- Sociodemographic and professional data: age, sex, years of experience, educational level, work setting, and population served.
- Ethics education: type and duration of training and perceived relevance of ethics in undergraduate and continuing education.
- Knowledge of the Code of Ethics: familiarity with its content, sources of information, perceived importance, and practical application.
- Ethical conflicts: frequency, nature, and strategies for addressing dilemmas.
- Perceptions of professional ethics: interest, attitudes, and perceived role of the Code in practice.

The instrument combined closed-ended questions (dichotomous and multiple choice) with five-point Likert scales to assess attitudes and perceptions. A pilot test was conducted with occupational therapists from different regions, allowing refinements to item wording and sequence to improve clarity and content validity.

### 2.4. Data Collection Procedure

Data were collected between March and September 2022 using an online questionnaire. The survey link was disseminated through professional organizations and occupational therapy networks, including professional social media channels (LinkedIn, Facebook, and specialized groups). Participants received study information before accessing the questionnaire, and informed consent was obtained via a mandatory checkbox at the beginning. To support data quality, technical and procedural controls were applied (e.g., restricting submissions to one response per device and screening for completeness prior to analysis).

Participants were provided with detailed information on the study objectives, voluntary participation, confidentiality of the data, and their right to withdraw at any time. Informed consent was obtained through a checkbox at the beginning of the questionnaire.

To ensure internal validity and reliability, several control mechanisms were implemented, including restricting responses to one submission per device and reviewing completeness before analysis.

### 2.5. Data Analysis

Data were processed using IBM SPSS Statistics v.24 (SPSS Inc., Chicago, IL, USA). Descriptive and inferential statistical analyses were applied to characterize the sample and examine associations among study variables.

First, descriptive statistics—frequencies, percentages, means, and standard deviations—were calculated for sociodemographic, educational, and ethical variables.

Subsequently, inferential analyses were conducted to explore relationships among different dimensions of the questionnaire:

- Pearson’s correlation coefficient (r) was used to identify linear relationships between continuous variables such as age, years of experience, level of knowledge of the Code, and interest in ethics.
- Chi-square tests (χ^2^) were applied to examine associations among categorical variables, including ethics training, participation in ethics committees, and presence of ethical conflicts.
- One-way analysis of variance (ANOVA) was performed to compare differences in knowledge of the Code based on educational level.

The statistical significance threshold was set at *p* < 0.05. Results were organized and presented in tables and figures following the guidelines of the 7th edition of the American Psychological Association (APA), including descriptive figures and correlation tables integrated within the manuscript.

### 2.6. Ethical Considerations

The study complied with the European Union General Data Protection Regulation (GDPR; Regulation (EU) 2016/679) and, subsequently, with Spanish data protection legislation, namely Organic Law 3/2018 on Personal Data Protection and the Guarantee of Digital Rights (LOPDGDD). It also adhered to the principles of the Declaration of Helsinki (World Medical Association, 2013). Ethical approval was obtained from the Research Ethics Committee of the Autonomous Community of Aragon (CEICA) (approval code: PI21/269). All data were processed confidentially and reported only in aggregate form, ensuring that individual participants could not be identified.

In addition to international guidelines and statutory requirements, the study was conducted in accordance with the Spanish Occupational Therapy Code of Ethics and Deontology approved by the Consejo General de Colegios de Terapeutas Ocupacionales (CGCTO, 2020), which includes specific provisions for research practice. In line with this framework, the research team upheld the applicable legal and ethical requirements for health research, ensured participant confidentiality throughout the study and after its completion, and sought independent ethical oversight through a competent research ethics committee.

## 3. Results

### 3.1. Sociodemographic and Professional Characteristics of the Sample

The final sample consisted of 596 occupational therapists practicing in Spain. Most participants were women (*n* = 516, 86.6%), followed by men (*n* = 73, 12.2%), and 1.2% preferred not to disclose their sex (*n* = 7). Regarding age, 38.1% were 20–30 years old, 38.8% were 31–40, 20.0% were 41–50, 2.5% were 51–60, and 0.7% were older than 60. In terms of professional experience, 52.0% had 0–10 years of experience, 34.1% had 11–20, 12.4% had 21–30, and 1.5% had more than 30 years of experience. Concerning educational level, 53.9% held a bachelor’s degree, 42.6% had a master’s degree, and 3.5% held a doctoral degree.

The most common work settings were hospital/clinical environments (28.9%), residential care facilities (26.5%), and day centers or outpatient services (19.6%). The remaining participants worked in home care (6.0%), higher education (3.4%), or other settings (15.6%), Table 1.

### 3.2. Ethics Education and Participation in Ethics Committees

A total of 65.3% of participants reported having completed a specific ethics course during their university studies; however, only 15.6% stated that the instructor was an occupational therapist. Additionally, 32% had received further training in ethics, deontology, or bioethics after graduation, while 66.6% had not taken any continuous education courses in these areas.

Only 25.3% worked in institutions with clinical or research ethics committees, and a mere 9.2% had participated in such committees—indicating limited access to formal ethics deliberation structures in Spanish occupational therapy workplaces, Table 2.

### 3.3. Knowledge and Perceived Importance of the Code of Ethics

Most participants (67.3%) considered the Occupational Therapy Code of Ethics to be “very important” for professional practice, and 25.7% rated it as “important.” However, 11.4% reported being unaware of its existence. Only 16.3% correctly identified the professional values included in the Code, and 41.9% could not identify the regulatory body that approved it, Table 3.

### 3.4. Ethical Conflicts in Professional Practice

Overall, 43.1% of participants reported having experienced ethical conflicts in their professional practice, while 50.8% indicated that they had not, and 6.1% did not respond. The conflicts most frequently reported were related to insufficient time to provide adequate care, scarcity of resources, management-imposed directives, and tensions between client autonomy and institutional policies.

From the total sample (*n* = 596), 262 participants reported having experienced ethical conflicts. Among them, 160 participants explicitly identified one or more types of ethical conflict using the categories provided in the questionnaire. The analysis presented below focuses exclusively on this subsample (*n* = 160) and provides a quantitative distribution of the main types of ethical conflicts identified, grouped according to their predominant ethical dimension (Table 4).

When experiencing an ethical conflict, the most common strategy was consulting the interdisciplinary team (25.5%), followed by discussions with other occupational therapists (24.3%). Only 4.5% used the Code of Ethics as a tool, and 2.7% consulted an ethics committee (Table 5).

### 3.5. Group Comparisons and Correlations

The comparisons of proportions (χ^2^) showed significant associations between ethical training and knowledge of the Code of Ethics (χ^2^ = 12.45, *p* = 0.002), as well as between participation in ethics committees and the presence of reported ethical conflicts (χ^2^ = 9.13, *p* = 0.010). In addition, the one-factor ANOVA revealed differences in the level of knowledge of the code according to the educational level achieved (F = 5.67, *p* = 0.004), being higher among those who had postgraduate studies. Likewise, the analysis of Pearson correlations revealed positive relationships between several dimensions of the study: age and years of experience showed a strong correlation (r = 0.72, *p* < 0.001); a moderate correlation was also identified between experience and knowledge of the Code of Ethics (r = 0.31, *p* = 0.008) and between ethical interest and continuing education (r = 0.36, *p* = 0.004). No significant correlations were found between age and the frequency of ethical conflicts (r = 0.08, *p* = 0.150), Table 6 and Table 7.

## 4. Discussion

The results of this study provide a broad and updated overview of the understanding and application of professional ethics among occupational therapists in Spain. Overall, the findings reveal a high valuation of professional ethics and a positive attitude toward ethical reflection; however, they also show significant shortcomings in systematic ethics training, knowledge of the Code of Ethics, and the use of institutional structures for moral support. These results are consistent with international literature describing the presence of structural ethical tensions and a gap between the values of the profession and the real conditions of clinical practice [2,3,4,7,10].

First, the demographic composition of the sample—predominantly young and female (Table 1)—is in line with the professional profile of occupational therapy in Europe [15]. Previous studies indicate that professionals with fewer years of experience tend to base their decisions on intuitive or contextual judgments rather than on explicit deontological references [5]. This may help explain the positive correlation observed between years of experience and knowledge of the Code of Ethics, as well as the significant differences associated with educational level: greater training and professional trajectory are associated with greater capacity to recognize and analyze ethically complex situations.

The results also reveal an asymmetry between the recognition of the importance of ethics and operational knowledge of the Code. While most participants considered the Code to be “very important” (Table 3), more than 40% could not identify the approving body, and only 16% knew its fundamental principles (Table 3). Moreover, when facing ethical conflicts, only a small proportion reported consulting the Code (4.5%) or an ethics committee (2.7%) (Table 4). This echoes the trends highlighted by Drolet [12] and Durocher [2], who argue that deontological codes often function as poorly internalized normative documents whose effectiveness depends on their integration into professional and educational culture. Thus, the detected gap suggests that in Spain the Code may operate more as a symbolic reference than as a practical tool for daily ethical deliberation.

Ethics education emerges as a key factor influencing knowledge and application of the Code. Although most participants reported having received some ethics education during university (65.3%; Table 2), only 15.6% indicated that the course was taught by an occupational therapist (Table 2), and a substantial proportion selected “Not sure/No answer” for this item or reported that it was not applicable, suggesting uncertainty about the disciplinary framing of ethics education. Importantly, ethics education may encompass distinct components: (i) conceptual and normative knowledge (principles, standards, and regulatory frameworks), (ii) deliberative competence (case-based reasoning, moral sensitivity, and justification), and (iii) institutional and collective conditions that enable ethical practice (e.g., structured deliberation spaces and organizational support). This does not fully align with the findings of Laborda [5], who noted that all curricula—except for one university—already included at least one course oriented toward ethics or deontology. These courses were often optional or basic, typically delivered early in the program, and assigned to various academic departments, most commonly Legal Medicine and Law. This disciplinary distribution is relevant because it shapes the theoretical and practical orientation of ethical content. Previous studies indicate that ethics education in occupational therapy tends to be theoretical, fragmented, and reliant on faculty interest [6,20]. The low prevalence of continuing ethics education observed in this study (Table 2) may help explain the predominance of informal strategies—such as discussions with colleagues—over the use of structured methodologies or institutional mechanisms for resolving ethical dilemmas.

Another relevant finding is the limited presence of and participation in ethics committees, with only 9.2% of therapists having been involved. This aligns with previous work by Rivard and Brown [9] and Durocher et al. [2], which shows that formal ethical deliberation is still limited in rehabilitation settings. The positive correlation between participation in committees and recognition of ethical conflicts suggests that involvement in such spaces increases moral sensitivity, consistent with Drolet’s concept of “situated ethics,” understood as a reflective process rooted in practice rather than a purely normative approach [16]. From this perspective, recognizing an ethical conflict is not a sign of professional weakness but an indication of ethical awareness and commitment to occupational justice.

The proportion of professionals reporting ethical conflicts (43.1%) is consistent with international findings. VanderKaay et al. [7] and Drolet [3] identified similar prevalence rates in hospital and community settings, where tensions commonly arise between autonomy, beneficence, and equity. In line with these findings, the distribution of conflicts by ethical dimension observed in this study (Table 4) shows a clear predominance of relational and structural conflicts, reinforcing the view that ethical challenges in occupational therapy are more often embedded in everyday interactions and organizational constraints than in isolated moral dilemmas.

The limited use of the Code of Ethics as a tool for resolving conflicts—consulted by only 4.5% of participants (Table 4)—underscores the gap between formal norms and lived practice. Likewise, only 2.7% reported consulting an ethics committee when facing ethical conflicts (Table 4), which is consistent with the limited access to such structures reported in Table 2. According to Drolet and Baril [4], therapists often rely on situated moral reasoning and peer dialog, especially when codes are not perceived as readily applicable to context-specific constraints. This aligns with Hudon’s description of “pragmatic ethical reasoning,” which prioritizes practical feasibility over literal adherence to abstract principles [5]. Taken together, these patterns suggest that institutional barriers, limited familiarity with formal resources, and the usability of normative documents in time-pressured contexts may contribute to the low reported use of the Code and ethics committees.

The correlation patterns further reinforce this interpretation: while experience and training were associated with knowledge of the Code, neither age nor experience alone predicted stronger ethical interest. This is consistent with the positions of Rivard [9] and Khaleghi et al. [8], who argue that ethical commitment depends more on reflexivity and organizational support than on the accumulation of years in practice. Strengthening ethical competence therefore requires not only disseminating normative frameworks but also fostering deliberative environments that support moral agency.

Taken together, the results confirm that ethics in occupational therapy is a practice under continuous construction, where critical reflection must contend with structural and cultural limitations. Following Drolet [16] and Durocher [2], professional ethics should be understood as a dialogical and contextualized process rather than a mere application of universal principles. In this regard, the present study provides empirical evidence that complements existing theoretical approaches, positioning the Spanish context within the broader international discussion on deontology and occupational justice.

In line with the findings, the following practical implications can be derived. First, the coexistence of a high level of symbolic endorsement of the Code with limited knowledge of its foundations suggests that merely disseminating the document as informational material is insufficient to foster meaningful professional appropriation. Accordingly, professional organizations should move from passive distribution strategies toward active implementation approaches, oriented to applied learning and the transfer of ethical principles into clinical practice.

Specifically, it would be advisable to design and deliver case-based learning workshops grounded in real and recurrent situations encountered in occupational therapy practice in Spain, in which the Code’s principles are addressed as operational tools for clinical reasoning, ethical deliberation, and decision-making. To maximize their utility, such workshops should be underpinned by a structured methodology tailored to the context and, where feasible, be facilitated by occupational therapists with training in applied ethics, thereby strengthening professional ownership of ethical guidance.

Furthermore, to enhance the accessibility and applicability of the content, the development of concise, practice-oriented resources (e.g., principle-based summaries, quick-reference guides, guiding questions, and clinical vignettes) is recommended to facilitate use in situations of ethical conflict. Taken together, these measures would help reduce the gap between declared adherence to the Code and practical knowledge of its principles, and they should be complemented by organizational mechanisms that enable deliberation (e.g., ethics rounds and clear referral pathways to ethics committees) to support ethically accountable practice across diverse settings.

This study presents certain methodological limitations. First, the cross-sectional design does not allow causal relationships to be established. Although the correlations identified reveal relevant associations—such as between ethics education and knowledge of the Code—it is not possible to infer causality, as the data correspond to a single time point.

Second, the use of non-probabilistic convenience sampling limits the representativeness of the findings. Despite broad participation (596 therapists from different regions), the sample may not fully reflect the actual distribution of occupational therapists in Spain, particularly given the predominance of women and younger professionals (Table 1). Moreover, data collection relied on an online questionnaire disseminated through professional associations and social networks, which may have introduced selection and self-selection biases; professionals more connected to these channels or with a prior interest in ethics may have been more inclined to participate. These factors may affect external validity and should be considered when interpreting prevalence estimates and associations.

A third limitation relates to the self-reported nature of the questionnaire, which may have introduced social desirability bias. Participants may have overstated their knowledge or ethical involvement. Nevertheless, anonymity reduces this risk, and such bias is common in ethics research. Finally, data were collected between March and September 2022. Although core ethical principles and the structural constraints shaping practice tend to be relatively stable, dissemination, uptake, and perceived usability of professional guidance may evolve over time due to professional initiatives, regulatory developments, or changes in service organization. Accordingly, the present findings should be interpreted as a snapshot of the Spanish context at that time and periodically reassessed through replication to monitor change; potential institutional or regional differences in the availability of ethical support may also have influenced responses.

Future research should incorporate qualitative methodologies—such as interviews, focus groups, or narrative analyses—to explore the lived ethical experiences of occupational therapists and the processes of moral reasoning guiding their decisions. Longitudinal studies are also recommended to examine how knowledge and internalization of the Code of Ethics evolve across different stages of professional development. Furthermore, evaluating the effectiveness of ethics training programs is essential to determine their impact on moral deliberation, decision-making quality, and prevention of moral distress. Finally, examining organizational and contextual factors that facilitate or hinder ethical deliberation in clinical and community settings is necessary. Together, these lines of inquiry will contribute to a more comprehensive and contextually grounded understanding of professional ethics, acknowledging its dynamic nature and dependence on institutional and sociocultural environments.

## 5. Conclusions

This national cross-sectional study provides updated empirical evidence on professional ethics in occupational therapy in Spain by examining knowledge and reported use of the Code of Ethics, the occurrence of ethical conflicts, and the strategies available to address them in practice. Overall, the findings indicate a consistent gap between the high value attributed to the Code as a normative reference and limited applied knowledge, suggesting that it often remains a symbolic or referential framework rather than a tool routinely integrated into clinical reasoning and decision-making in concrete situations.

Ethical conflicts emerge as relatively frequent experiences in professional practice and are predominantly associated with relational dynamics (tensions in interactions with service users and families) and structural conditions of work (organizational constraints, workload and performance pressures, and resource availability), alongside challenges related to professional autonomy and interprofessional coordination. When responding to these conflicts, practitioners tend to rely primarily on informal, situated resources—such as consultation within the interdisciplinary team or with occupational therapy peers—whereas formal mechanisms for ethical support and deliberation are used to a clearly lesser extent, consistent with the limited availability or accessibility of specific institutional structures.

In terms of implications, the results support strengthening ongoing, practice-oriented, case-based ethics education that goes beyond normative dissemination and fosters deliberative, argumentative, and justificatory competencies. In parallel, it is essential to develop and consolidate institutional supports that enable ethical praxis in real-world settings: formal deliberation spaces, clear pathways for ethics consultation, and organizational conditions that make it feasible to implement ethically justified decisions. Future research should complement this approach with qualitative and longitudinal designs to clarify the mechanisms that generate and sustain ethical conflicts, examine variation across practice settings, and evaluate the effects of educational and institutional interventions on the effective use of the Code and the ethical sustainability of professional practice.

## Figures and Tables

**Table 1:** Sociodemographic and professional characteristics of participants.

Variable	Category	*n*	%
Sex	Woman	516	86.6
	Man	73	12.2
	Other	7	1.2
Age (years)	20–30	227	38.1
	31–40	231	38.8
	41–50	119	20.0
	51–60	15	2.5
	>60	4	0.7
Years of experience	0–10	310	52.0
	11–20	203	34.1
	21–30	74	12.4
	>30	9	1.5
Education level	Bachelor’s degree	321	53.9
	Master’s degree	254	42.6
	Doctoral degree	21	3.5
Primary work setting	Hospital/Clinic	172	28.9
	Residential center	158	26.5
	Day center/Outpatient	117	19.6
	Home care	36	6.0
	University	20	3.4
	Other	93	15.6

**Table 2:** Ethical training and participation in ethics committees.

Variable	Category	*n*	%
Ethics course during university	Yes	389	65.3
	No	186	31.2
	Not sure/No answer	21	3.5
Course taught by an occupational therapist	Yes	93	15.6
	No	287	48.2
	Not sure/No answer	39	6.5
	Not applicable (no ethics course)	174	29.2
	Missing	3	0.5
Postgraduate ethics training	Yes	191	32.0
	No	397	66.6
	Not sure/No answer	8	1.3
Workplace has ethics committee	Yes	151	25.3
	No	324	54.4
	Not sure/No answer	121	20.3
Participation in ethics committees	Yes	55	9.2
	No	541	90.8

**Table 3:** Knowledge and perceived importance of the code of ethics.

Variable	Category	*n*	%
Importance of the Code	Very important	401	67.3
	Important	153	25.7
	Neutral	35	5.9
	Not important	7	1.2
Aware of the Code’s existence	Yes	528	88.6
	No	68	11.4
Identifies CGCTO as approving body	Yes	346	58.1
	No	250	41.9
Knows the professional values contained in the Code	Yes	97	16.3
	No	499	83.7

**Table 4:** Distribution of ethical conflicts by predominant ethical dimension (*n* = 160).

Ethical Dimension	Type of Ethical Conflict	*n*	%
Relational	Conflicts with clients	68	42.5
	Conflicts with family members/caregivers	61	38.1
Structural	Institutional constraints	58	36.3
	Productivity pressure	49	30.6
	Lack of resources/workload overload	44	27.5
	Regulatory gaps/lack of ethical guidance	29	18.1
Professional	Lack of professional autonomy	46	28.8
	Interprofessional conflicts	39	24.4
	Competence-related issues	37	23.1
	Moral distress	35	21.9

Note: Categories are not mutually exclusive; a single participant could report more than one type of ethical conflict.

**Table 5:** Ethical conflicts and resolution strategies.

Variable	Category	*n*	%
Has faced ethical conflicts	Yes	262	43.1
	No	308	50.8
	Not sure/No answer	37	6.1
Main strategy used	Consult interdisciplinary team	155	25.5
	Consult OT colleagues	148	24.3
	Report to supervisor	81	13.4
	Consult Code of Ethics	27	4.5
	Consult ethics committee	16	2.7
	Solve individually/No action	8	1.8

**Table 6:** Group comparisons.

Comparison (χ^2^ and ANOVA)	Statistic	df	*p*	Interpretation
Ethical training—Knowledge of the code	χ^2^ = 12.45	2	0.002	Significant association
Participation in committees—Presence of conflicts	χ^2^ = 9.13	1	0.010	Significant association
Educational level—Knowledge of the code	F = 5.67	2	0.004	Significant difference

**Table 7:** Correlations.

Variables (Pearson Coefficient)	r	*p*	Interpretation
Age—Years of experience	0.72	<0.001	Strong positive correlation
Experience—Knowledge of the code	0.31	0.008	Moderate positive correlation
Ethical interest—Continuing education	0.36	0.004	Moderate positive correlation
Age—Ethical conflicts	0.08	0.150	Not significant

## Data Availability

The raw data supporting the conclusions of this article will be made available by the authors on request.
